# Editorial: Translational Research in Thyroid Cancer

**DOI:** 10.3389/fendo.2020.00224

**Published:** 2020-04-15

**Authors:** Vasyl Vasko, Electron Kebebew, Sheue-yann Cheng, Kirk Ernest Jensen

**Affiliations:** ^1^Department of Pediatrics, Uniformed Services University of the Health Sciences, Bethesda, MD, United States; ^2^Department of Surgery, Stanford University, Stanford, CA, United States; ^3^Laboratory of Molecular Biology, National Cancer Institute, Bethesda, MD, United States

**Keywords:** thyroid cancer, stem cells, cell signaling, FNA (fine needle aspiration), reactive oxygen species

Advances in high-throughput molecular technologies have improved our understanding of the molecular changes associated with thyroid cancer initiation and progression. The translation into clinical use, based on molecular profiling of thyroid tumors, has allowed a significant improvement in thyroid cancer diagnosis, patient risk stratification and in the identification of targets for precision therapy. This Research Topic, “Translational Research in Thyroid Cancer” contains a collection of studies that address emerging questions in thyroid cancer biology, and details approaches that leverage genomic-based algorithms to improve thyroid cancer diagnosis.

The evolution of cancer is not solely dictated by an individual tumor cell's irregular proliferation conferring gains in somatic mutations. A tumor's microenvironment, along with individual cancer cell genome instability, encourages clonal expansion, and heterogeneity. McGonagle and Nucera write a timely review discussing how novel experimental models will enhance our understanding of clonal and sub-clonal reconstruction and tumor evolution exposed to treatments during ultra-precision targeted therapies.

In keeping with this theme, the research article of Kim and Koo addresses the role of cancer stem cells in thyroid cancer. Cancer stem cells (CSCs) have important roles in cancer development, growth, recurrence, and metastasis owing to their potential to self-renew and differentiate into various cells lineages. This study highlights how the overexpression of CSC markers in thyroid cancer tissue is associated with shorter progression-free survival. It also demonstrates that staining of CSC markers may provide useful information for predicting patient outcomes.

Several studies in this collection examine associations between genomic abnormalities and cell signaling activation in thyroid cancer. Kari et al. explore molecular mechanisms implicated in development of follicular thyroid cancer (FTC). The authors have examined a mouse model for FTC caused by tissue-specific ablation of the Protein Kinase A (PKA) regulatory subunit, Prkar1a, either by itself or in combination with knockout of Pten. To understand the mechanism by which PKA activates mTOR, the authors examined intracellular kinases known to modulate mTOR function. Although AMP-activated kinase (AMPK) has been characterized as a negative regulator of mTOR activity, this tumor model exhibited activation of both AMPK and mTOR. Because the AMPK/LKB1 pathway has traditionally been considered a tumor suppressor, results of this study indicates that it may have a more complex role in the thyroid gland.

Reactive oxygen species (ROS), such as hydrogen peroxide (H_2_O_2_), are required for normal thyroid cell proliferation as well as for synthesis of the main hormones secreted by thyroid follicular cells. Thyroid follicular cells need to protect themselves against oxidative stress, and one such protective mechanism is the antioxidant response pathway centered on the nuclear factor erythroid 2-related transcription factor 2 (Nrf2). Renaud et al. contributed an article reviewing the involvement of Nrf2 signaling in thyroid physiology and particularly in thyroid cancer.

The review paper submitted by Ramírez-Moya and Santisteban focuses on understanding the functional significance of the most up- or down-regulated miRNAs in thyroid cancer on the main signaling pathways hyperactivated in this tumor type. Specifically, this study discusses the major miRNAs targeting proteins of the MAPK, PI3K, and TGFβ pathways in thyroid cancer. Given the importance of miRNAs in cancer as diagnostic, prognostic, and therapeutic candidates, a better understanding of this cross-talk might shed new light on the treatment of thyroid cancer.

Aberrant gene methylation plays an important role in human tumorigenesis, including thyroid tumorigenesis. Liu et al. performed an analysis of the methylation status of cellular retinoic acid binding protein 2 (CRABP2) and the expression patterns of methylation enzymes (DNMT1, DNMT3A, and DNMT3B). This study emphasizes that resveratrol, a non-toxic polyphenol compound, exerts demethylation in the same way as gemcitabine, and can be considered as an alternative demethylating drug for cancer prevention and treatment.

Though less frequent than the adult type, differentiated thyroid cancer (DTC) can also develop during childhood. The differences in the clinical and pathological features of pediatric vs. adult thyroid cancer could relate to a different genetic profile. In this Research Topic, Galuppini et al. characterize pediatric and adult thyroid cancers focusing on clinical features and outcomes; molecular profile, with particular reference to the study of point mutations of the *BRAF, RAS, TERT* genes, and *RET/PTC* translocations; and correlations between clinical and molecular findings. This study underscores how pediatric DTC is clinically more aggressive at diagnosis and more likely to recur than its adult counterpart. Unlike the adult disease, point mutations have not been demonstrated to have a key genetic role.

Fine needle aspiration (FNA) cytology, a diagnostic test central to thyroid nodule management, may yield indeterminate results in up to 30% of cases. In this Research Topic, two studies address the diagnostic utility of the Afirma genomic tests in the evaluation of thyroid nodules. Hao et al. examine the analytical robustness and reproducibility of the Afirma Genomic Sequencing Classifier test. The work of Angell et al. assesses the analytical and clinical validation of the Afirma Xpression Atlas (XA), which detects gene variants and fusions from a curated panel of 511 genes via dedicated FNA samples using whole-transcriptome RNA-sequencing. These studies show that genomic information provided by the Afirma test may inform clinical decision-making with precision medicine insights across a broad range of FNA sample types encountered in the care of patients with thyroid nodules and thyroid cancer.

This original series of articles details emerging approaches in thyroid cancer research and applications of molecular-based algorithms for diagnosis of thyroid cancer.

## Author Contributions

All authors listed have made a substantial, direct and intellectual contribution to the work, and approved it for publication.

### Conflict of Interest

The authors declare that the research was conducted in the absence of any commercial or financial relationships that could be construed as a potential conflict of interest.

